# Mechanism and protective properties of S-nitrosation by nitric oxide in cardiac mitochondria

**DOI:** 10.1186/2050-6511-16-S1-A21

**Published:** 2015-09-02

**Authors:** Thomas Krieg

**Affiliations:** 1 Department of Medicine, University of Cambridge, UK

## 

Oxidative damage from elevated production of reactive oxygen species (ROS) contributes to ischemia-reperfusion injury (IRI) in many pathologies, such as myocardial infarction, stroke, and organ transplantation. We recently determined a critical mechanism for protection from IRI by nitric oxide (NO)-dependent S-nitrosation using a mitochondria-selective S-nitrosating agent, MitoSNO. MitoSNO contains an S-nitrosating moiety derived from the NO-donor SNAP, attached to a lipophilic triphenylphosphonium cation that leads to its rapid and extensive uptake within mitochondria in vivo. Protection by MitoSNO is afforded by inhibition of complex I through selective S-nitrosation of Cys39 on the ND3 subunit, which becomes susceptible to modification only after ischaemia. The observed posttranslational alteration of complex I slows the reactivation of mitochondria during the crucial first minutes of the reperfusion of ischemic tissue, thereby decreasing ROS production, oxidative damage and tissue necrosis.

Furthermore, we could show that the reperfusion-induced ROS production is not an unspecific event of complex I reactivation but is highly regulated by a dramatic increase in succinate during ischaemia. ROS are subsequently produced via reverse electron transport and inhibition of the succinate rise during ischaemia proved to be a further promising target to protect against IRI injury.

Taken together, reversible S-nitrosation of complex I or the prevention of ischaemic succinate accumulation results in significant protection from acute and long-term effects of IRI and provide evidence for compounds selectively targeting the mitochondria, such as MitoSNO, to be a promising therapeutic strategy for translation to use in humans.

